# The complete mitochondrial genome and phylogenetic analysis of *Lotus corniculatus* (Fabaceae, Papilionoideae)

**DOI:** 10.3389/fpls.2025.1555595

**Published:** 2025-03-11

**Authors:** Xiaofei Chen, Zinian Wu, Yanting Yang, Qibo Tao, Na Na, Wenya Wan, Chunyu Tian, Wenlong Gong, Zhiyong Li

**Affiliations:** ^1^ College of Grassland Science, Qingdao Agricultural University, Qingdao, China; ^2^ Institute of Grassland Research, Chinese Academy of Agricultural Sciences, Hohhot, China; ^3^ Key Laboratory of Grassland Resources and Utilization of Ministry of Agriculture, Ministry of Agriculture of the People's Republic of China, Institute of Grassland Research, CAAS, Hohhot, China

**Keywords:** *Lotus corniculatus*, mitochondrial genome, gene transfer, RNA editing, phylogenetic analysis

## Abstract

**Introduction:**

*Lotus corniculatus* is a perennial leguminous herb and serves as a high-quality forage, playing a key role in both grassland ecological restoration and the development of grazing livestock farming.

**Methods:**

In this study, we successfully assembled the *L. corniculatus* mitochondrial genome and investigated various related aspects, including genomic features, RNA editing sites, codon preference, gene transfer events, and phylogeny.

**Results and discussion:**

We found that the length of the *L. corniculatus* mitochondrial genome is 401,301 bp, and its GC content is 45.15%. It consists of 53 genes, comprising 32 protein-coding genes, 3 ribosomal RNA genes, and 18 transfer RNA genes. A total of 146 scattered repeats, 8 tandem repeats, and 124 simple sequence repeats are present in the mitochondrial genome. A thorough examination of all protein-coding genes revealed 485 instances of RNA editing and 9579 codons. Additionally, 57 homologous fragments were identified in *L. corniculatus* mitochondrial genome and chloroplast genomes, accounting for approximately 4.04% of the *L. corniculatus* mitochondrial genome. Furthermore, a phylogenetic tree based on mitochondrial genome data from 33 species belonging to four Fabaceae subfamilies and two species from other families validated the evolutionary relationship of Lotus. These findings have significant implications for understanding the organization and evolution of the *L. corniculatus* mitochondrial genome as well as for the identification of genetic markers. They also offer valuable perspectives relevant to devising strategies for molecular breeding and evolutionary categorization of legumes.

## 1 Introduction

*Lotus corniculatus*, a perennial leguminous herb native to warm regions of Eurasia, is now distributed widely across Europe, North and South America, India, Australia, and New Zealand (Xu, 1988). *L. corniculatus* is an excellent forage, possesses several benefits, and has diverse uses, which highlight its economic value. Owing to its rich nutritional content, palatability, and ecological adaptability, *L. corniculatus* is extensively used in the livestock industry and ecological restoration (Li et al., 2024). It can also be utilized as a soil remediation material, with a well-developed root system that enhances soil organic matter and nitrogen levels, improving overall soil quality (Zhao et al., 2013). Moreover, its strong resistance to barren conditions makes it suitable for landscaping purposes, such as soil conservation and prevention of erosion (Du, 2022). However, *L. corniculatus* is susceptible to various stressors, which impede its growth and development and can even lead to plant death, significantly reducing the yield (Li et al., 2020). The development of more resilient *L. corniculatus* species could help to mitigate this problem. However, traditional breeding has some disadvantages, such as long cycles, difficult cross operation, low fruit setting rate and self-cross decline. In recent years, with the rapid development of molecular biology, the use of molecular breeding technology to screen for good traits of *L. corniculatus* can not only significantly improve the breeding efficiency but also greatly shorten the breeding cycle, thus becoming an important means for improved *L. corniculatus* breeding. In addition, despite significant advances in molecular biology, genetics, and bioinformatics, studies of the molecular mechanism and evolution of *L. corniculatus* still lack adequate genomic information. The expansion of genetic resources can provide more accurate technical support for *L. corniculatus* in relation to gene mining, functional analysis and molecular marker breeding. Therefore, it is imperative to develop the genomic resources of *L. corniculatus*, a plant species of significant economic and practical value, in order to elucidate its evolutionary relationships and establish a solid foundation for further molecular investigations. At present, the chloroplast genome of *L. corniculatus* has been reported and lacks a mitochondrial genome to form a complete organelle genome resource.

Mitochondria are organelles essential for respiration and are the main producers of energy. Regarding their structure, mitochondria consist of two distinct membranes: an inner membrane that is extensively folded into cristae to maximize surface area, and an outer membrane that exhibits higher permeability and contains various transport proteins (Newton, 1988). The mitochondrial genome is important for energy metabolism and nucleocytoplasmic interactions within organisms, and has been prominently focused upon in evolutionary biology, genomics, bioinformatics, among other research domains (Clifton et al., 2004; Picault et al., 2004; Wang et al., 2020). Plant mitochondrial genomes exhibit polymorphisms, heterogeneity, complexity, and variability (Kubo and Newton, 2008). Compared with animal mitochondrial genomes, plant mitochondrial genomes are quite different in size, sequence composition and functional gene arrangement (Gualberto and Newton, 2017; Gualberto et al., 2014). The majority of plant mitochondrial genomes consist of circular double-stranded DNA, with sizes ranging from thousands to millions of base pairs. The length and size of mitochondrial genome sequences exhibit variability across different plant species (De Oca Balderas, 2021; Richardson et al., 2013; Wang et al., 2024b). The genome size of plant mitochondria exhibits considerable variability, yet the majority of mitochondrial protein-coding genes remain highly conserved, comprising primarily 24 core conserved genes and 17 variant genes (Møller et al., 2021). The plant mitochondrial genomes also contain a substantial amount of repetitive sequences, which can result in structural rearrangements of the mitochondrial genome and give rise to gene chimeras. This phenomenon renders the mitochondrial genome as a carrier of male sterility factors, thereby impacting plant survival (Zhong et al., 2021). Additionally, gene fragments originating from chloroplasts are universally present in the mitochondrial genomes of higher plants, and this migration event constitutes a pivotal aspect for investigating plant evolution (Turmel et al., 2016; Wang et al., 2024a). The nuclear genome is inherited from both parents, whereas the chloroplast and mitochondrial genomes are exclusively maternally inherited. This genetic mechanism eliminates paternity influence, facilitating the acquisition of genetic information, reducing the complexity of genetic research, and aiding in inferring phylogenetic relationships within or between species (Sato and Sato, 2013). The acquisition of genetic information from the mitochondrial genome, capitalizing on its smaller size compared to the nuclear genome, offers novel insights into physiological and biochemical changes during plant development, thereby enhancing information mining capabilities. Such information guides strategies for further exploration of the nuclear genome (Goremykin et al., 2009).

The rapid development of high-throughput sequencing technology has greatly promoted the research of mitochondrial genomics, and the methods of constructing pedigree relationships among different populations based on mitochondrial genome sequences have rapidly evolved. In recent years, the mitochondrial genomes of many legumes such as *Vigna radiata* (Alverson et al., 2011), *Vicia faba* (Negruk, 2013), *Glycine max* (Chang et al., 2013), and *Astragalus membranaceus* (Zhang et al., 2024) have been successfully sequenced and assembled. Therefore, remarkable progress has been made in the study of mitochondrial genome of legumes in terms of size, gene content and phylogenetic analysis (Feng et al., 2019). As of September 2024, despite the complete mitochondrial genomes of approximately 96 legume species being reported in NCBI, this number remains insufficient given the vast diversity of the legume family. Therefore, sequencing and assembling additional legume mitochondrial genomes can facilitate a deeper exploration of gene transfer mechanisms, gene content and function, changes in genome size and structure, interactions with other genomes, and applied research. This will enhance our understanding of the evolutionary processes and adaptability of legumes, while providing a theoretical foundation and technical support for genetic improvement and agricultural production.

In the present study, we used a combination of NovaSeq 6000 and PromethION sequencing methodologies to construct the mitochondrial genome of *L. corniculatus*. We identified diverse genomic characteristics, such as gene composition, RNA editing locations, and codon utilization patterns, within the *L. corniculatus* mitochondrial genome. Additionally, transfer events between *L. corniculatus* organelle genomes were thoroughly analyzed, and the phylogenetic relationships among different mitochondrial genomes were investigated in depth. This extensive investigation offers valuable genetic insights for further examination of the evolutionary and functional characteristics of *L. corniculatus* and should enhance our understanding of organelle genomes in legumes.

## 2 Materials and methods

### 2.1 Extraction of DNA, genome sequencing, and genome assembly

The *L. corniculatus* samples were collected from Hohhot, Inner Mongolia (40.57°N, 111.93°E), and stored in the National Perennial Forage Germplasm Resource Nursery (Hohhot, China). Fresh leaves were harvested, rinsed with ultrapure water, and promptly frozen in liquid nitrogen. They were stored in a deep freezer at −80°C for maintaining their integrity. DNA of *L. corniculatus* was extracted and purified using the CTAB method and Qiagen Blood & Cell Culture DNA Kit (Cat. no. 13323), respectively. Nanodrop results showed that the sample concentration was 140.9 ng/μL and the A260/A280 ratio was 1.82. Qubit test results showed that the sample concentration was 151 ng/μL. The ratio of Nanodrop to Qubit was 0.93. Sequencing was then performed using the Novaseq6000 (Illumina, San Diego, CA, USA) and PromethION (Oxford Nanopore Technologies, Oxford, UK) platforms. The Illumina NovaSeq 6000 generated a total of 51.64 million reads, corresponding to 14.52 GB of high-quality clean data. Concurrently, the Oxford Nanopore PromethION produced 1.26 million reads, yielding 15.45 GB of clean data. For mitochondrial genome assembly, the Nanopore long-read data were initially aligned against reference gene sequences using minimap2 v2.1 (Li et al., 2016). Subsequently, Canu v2.0 (Koren et al., 2017) was used for error correction and Bowtie2 v2.3.5.1 (Langmead and Salzberg, 2012) was employed to align the second-generation data with the corrected sequence. Finally, Unicycler v0.4.8 with default parameter sets was employed to merge the matched second-generation data with the corrected third-generation data to obtain a complete and accurate mitochondrial genome sequence. Visualization and manual adjustment of the splicing results were achieved using Bandage v0.8.1 (Wick et al., 2015). To ensure the reliability of the assembled mitochondrial genome, we conducted a comprehensive validation through coverage depth analysis using raw sequencing data from both NovaSeq 6000 and PromethION platforms. For the NovaSeq 6000 short-read data, we performed reference sequence alignment using Bowtie2 v2.3.5.1 (Langmead and Salzberg, 2012), which yielded a robust mean coverage depth of 361.33× across the mitochondrial genome (**Supplementary Figure S1**). In parallel, we processed Oxford Nanopore long-read data through reference-guided assembly with minimap2 v2.1 (Li et al., 2016), achieving a consistent mean coverage depth of 357.88× (**Supplementary Figure S2**). Both alignment procedures were complemented by SAMtools v1.9 (Li et al., 2009) for in-depth coverage analysis and quality assessment throughout the entire mitochondrial genome sequence. Sequences of the *L. corniculatus* mitochondrial genome were deposited in the GenBank (accession number: PP706441).

### 2.2 Genome annotation

GeSeq (Tillich et al., 2017) was used to annotate the *L. corniculatus* mitochondrial genome, employing the genome of closely related *L. japonicus* (NC_016743.2) as a reference. tRNAscan-SE (Chan et al., 2021) was employed to annotate the tRNA genes, and subsequent manual adjustments and corrections were made to refine the annotations of the mitochondrial genomes. Genome mapping was conducted using the Organellar Genome Draw (Greiner et al., 2019) software.

### 2.3 Repeat sequence identification

Scattered repeats were identified using the REPuter (Kurtz et al., 2001) program with the parameter settings: minimum repetition size, 30 base pairs (bp); Hamming distance, 3; unacceptable e value, 1e-5. The forward and reverse order, and palindromic and complementary repeats were identified. The Tandem Repeats Finder v4.09 (Benson, 1999) software was used to detect tandem repeats. Simple sequence repeats (SSRs) were identified using the MISA v2.1 (Thiel et al., 2003) software, employing the following parameters: single nucleotide = 10; dinucleotide = 5; trinucleotide = 4; tetranucleotide = 3; pentanucleotide = 3; and hexanucleotide = 3.

### 2.4 Analysis if the codon usage bias

Coding sequences (CDS) were extracted from the mitochondrial genomes using Genious Prime 2024.0.5 (Han et al., 2024). CodonW v1.4.2 (Peden, 2000) was used to perform statistical analysis of the codon usage of protein-coding genes (PCGs), and the relative synonymous codon usage (RSCU) was computed.

### 2.5 Prediction of RNA editing sites

Potential RNA editing sites in mitochondrial PCGs were identified using the SRA database (https://www.ncbi.nlm.nih.gov/sra/; accession numbers SRR11487696, SRR11487697, SRR11615684, SRR1161568, and SRR11615686). Dataset quality control was performed using the manual of REDO (Wu et al., 2018) software along with the trimmomatic 0.39.jar filter (Bolger et al., 2014). The BWA v0.7.15 (Li and Durbin, 2010) software was used to compare and locate the transcriptome sequencing results, filter out potential false-positive regulatory sites, and use PCGs as a reference. Single nucleotide polymorphisms (SNPs) were named using the SAMtools v1.17 (Li et al., 2009) and BCFtools v1.17 (Danecek et al., 2021), and RNA editing sites were identified and annotated using the REDO (Wu et al., 2018) software. Furthermore, SNPs were activated and RNA editing sites found inside genomic SNPs were removed using the BCFtools v1.17 (Danecek et al., 2021).

### 2.6 Identification of mitochondrial plastid DNAs

Homologous fragments in the mitochondrial and chloroplast genomes were identified using BLASTn v.2.14.1+ (Chen et al., 2015b), and the MTPTs results were visualized using TBtools-II v2.136 (Chen et al., 2023).

### 2.7 Phylogenetic analysis

We used the mitochondrial genome sequence of *L. corniculatus* obtained in this study and those of Fabaceae (**Supplementary Table S1**) retrieved from the NCBI database for phylogenetic analysis. The common genes of 33 *Fabaceae* species and two outgroups were extracted using the PhyloSuite v1.2.3 (Zhang et al., 2020a) software, and aligned with MAFFT v7.525 (Katoh et al., 2019). Disordered positions and divergent regions were removed using Gblocks 0.91b (Talavera and Castresana, 2007). The Model Finder (Kalyaanamoorthy et al., 2017) tool in PhyloSuite v1.2.3 (Zhang et al., 2020a) was used to select optimal partitions for Bayesian and ML constructions. Subsequently, the MrBayes v3.2.7 software (Ronquist et al., 2012) was used to construct a Bayesian Inference (BI) tree, with the optimal model being GTR+I+F. RAxML v8.2.12 (Stamatakis, 2014) was employed to construct the maximum likelihood (ML) tree with 1000 bootstrap replicates, and the optimal partition model was set as GTRGAMMAI.

### 2.8 Synteny analyses

The mitochondrial genomes of three legume species, *Lotus japonicus* (NC_016743.2), *Medicago truncatula* (NC_029641.1) and *Glycine max* (NC_020455.1), which are closely related to *L. corniculatus*, were selected from the NCBI database for synteny analysis. Firstly, the homologous sequences of these four species were pairwise compared using BlastN (Chen et al., 2015b) 2.13.0, and homologous sequences with a length of more than 300 bp were selected as collinear blocks. Subsequently, multicollinear maps were constructed using MCScanX (Wang et al., 2012).

## 3 Results

### 3.1 Structural characteristics of the *L. corniculatus* mitochondrial genome

**Figure 1:**
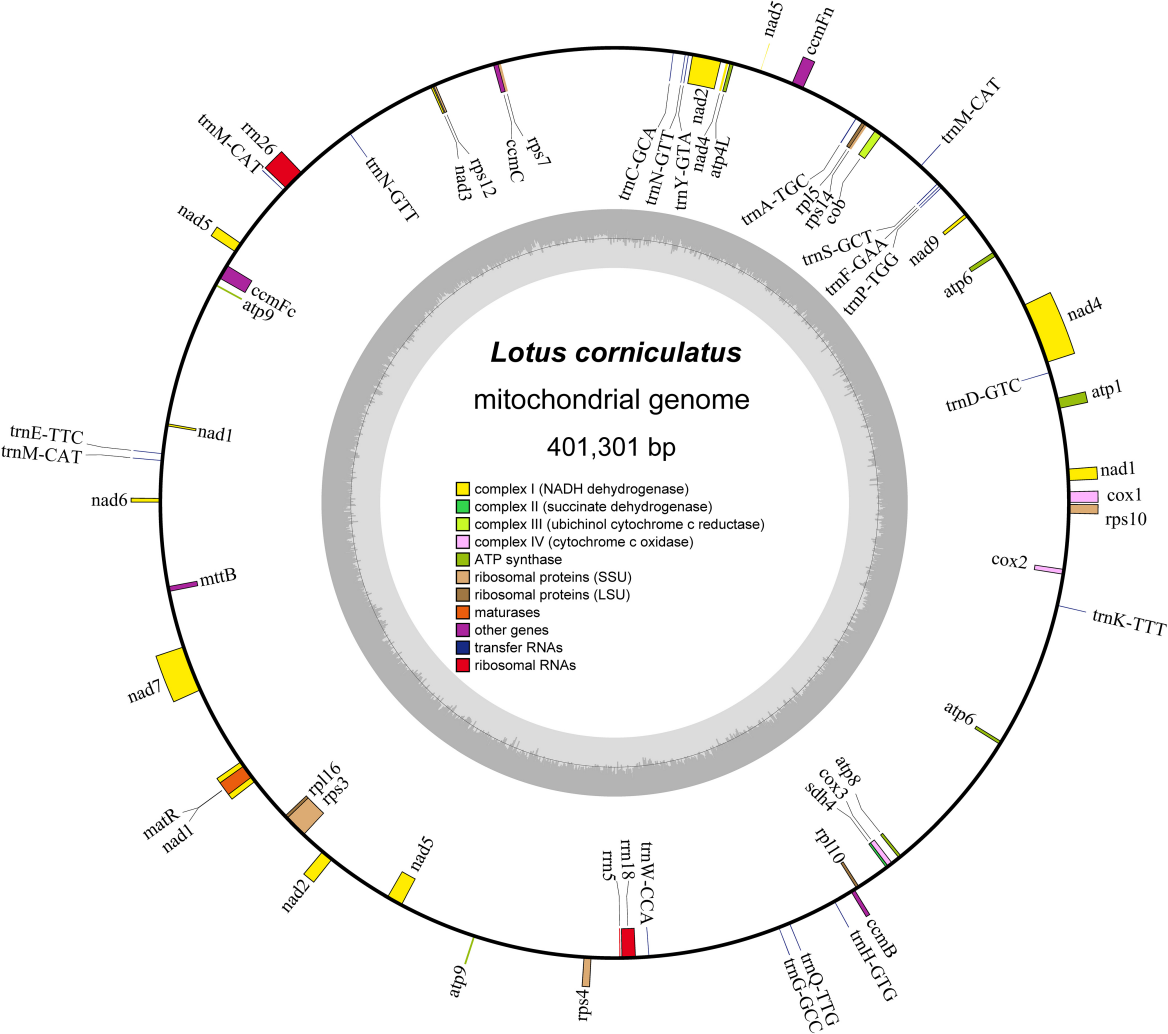
Schematic map of *Lotus corniculatus* mitochondrial genome structure. Genes positioned outside the outer circle represent forward transcribed genes, whereas those located inside the outer circle represent reverse transcribed genes. The region of the inner ring in carbon gray represents the GC concentration, and distinct groups of functional genes are indicated in varying hues.

The mitochondrial genome of *L. corniculatus* has a single master circular structure (Wu et al., 2022) of 401,301 bp with 45.15% GC content. The length of the CDS, tRNA, and rRNA is 28,962, 1,335, and 5,266 bp, and the GC content is 42.92%, 50.94%, and 51.73%, respectively (**Figure 1**). A total of 32 CDS were identified, which comprised 17 variable and 15 core genes. The core genes identified were one panthenol cytochrome c reductase gene (*cob*), three cytochrome c oxidase genes (*cox1*, *cox2*, and *cox3*), one mature enzyme gene (*matR*), five ATP synthetase genes (*atp1*, *atp4*, *atp6*, *atp8* and *atp9*), and four cytochrome c biogenesis genes (*ccmB*, *ccmC*, *ccmFc*, and *ccmFn*). A pseudogene, *atp6*, was additionally identified. The variable genes were one transport membrane protein (*mttB*), nine NADH dehydrogenase genes (*nad1*, *nad2*, *nad3*, *nad4*, *nad4L*, *nad5*, *nad6*, *nad7*, and *nad9*), two large ribosomal protein subunits (*rpl16* and *rpl5*), and five small ribosomal protein subunits (*rps10*, *rps12*, *rps14*, *rps3*, and *rps4*). Three pseudogenes were identified (*rpl10*, *rps7*, and *sdh4*). The genome comprises three rRNA and 18 tRNA genes (**Table 1**). The majority of the core, rRNA, variable, and tRNA genes were present in single copies. However, two copies of *atp6* and *trnN-GTT* and three of *trnM-CAT* are present (**Table 1**). The longest gene, *matR*, spans a length of 1,980 bp and encodes a protein with 660 amino acids. In contrast, the shortest gene, *atp9*, measures only 225 bp in length and codes for only 75 amino acids (**Supplementary Table S2**). The total number of genes analyzed was 53, of which nine were found to possess introns; these nine genes can be categorized as follows: *ccmFc*, *rps10*, *rps3*, and *trnA-TGC* each possessing a single intron; *nad4*, which contains three introns; and *nad1*, *nad2*, *nad5*, and *nad7* each possessing four introns (**Table 1**; **Supplementary Table S3**).

**Table 1:** Genetic composition of functional gene groups in the *Lotus corniculatus* mitochondrial genome.

	Group of genes	Gene name
Core genes	ATP synthase	#*atp6*, *atp1*, *atp4*, *atp6*, *atp8*, *atp9*(2)
	Cytochrome c biogenesis	*ccmB*, *ccmC*, ccmFc*, *ccmFn*
	Ubiquinol cytochrome c reductase	*Cob*
	Cytochrome c oxidase	*cox1*, *cox2*, *cox3*
	Maturases	*matR*
Variable genes	Transport membrane protein	*mttB*
	NADH dehydrogenase	nad1****, nad2****, *nad3*, nad4***, *nad4L*, nad5****, *nad6*, nad7****, *nad9*
	Ribosomal proteins (LSU)	#*rpl10*, *rpl16*, *rpl5*
	Ribosomal proteins (SSU)	#*rps7*, rps10*, *rps12*, *rps14*, rps3*, *rps4*
	Succinate dehydrogenase	*#sdh4*
rRNA genes	Ribosomal RNAs	*rrn18*, *rrn26*, *rrn5*
tRNA genes	Transfer RNAs	trnA-TGC*, *trnC-GCA*, *trnD-GTC*, *trnE-TTC*, *trnF-GAA*, *trnG-GCC*, *trnH-GTG*, *trnK-TTT*, *trnM-CAT(3)*, *trnN-GTT(2)*, *trnP-TGG*, *trnQ-TTG*, *trnS-GCT*, *trnW-CCA*, *trnY-GTA*

*Intron number (*: One introns; ***: Three introns; ****: Four introns); #Gene, Pseudo gene; Gene(2), Number of copies of multi-copy genes.

### 3.2 Anatomization of repeat sequences

**Figure 2:**
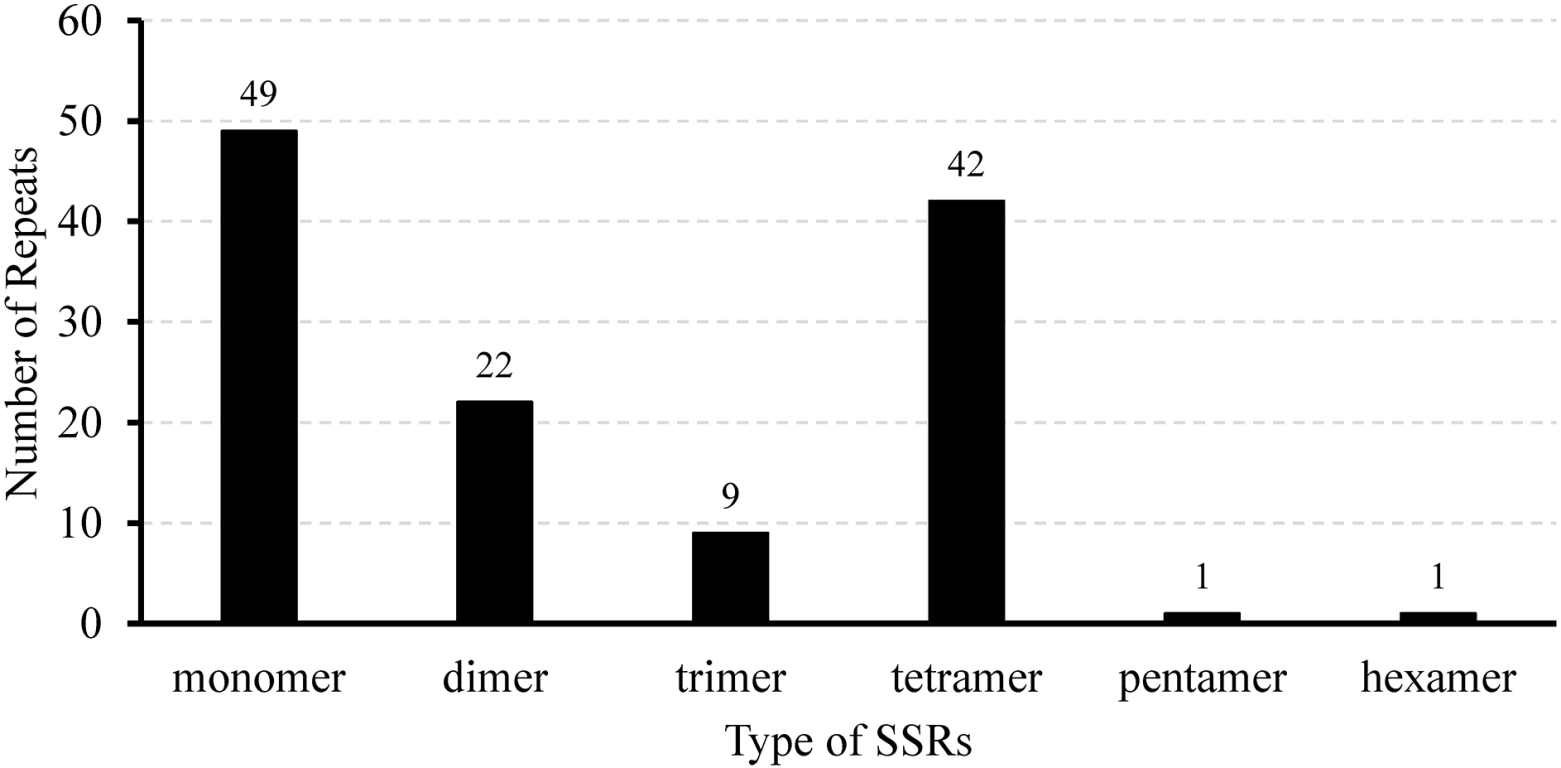
Quantitative distribution of different simple sequence repeat types in the *Lotus corniculatus* mitochondrial genome.

We identified 124 SSRs, which are 10–18 bp long tandem repeat sequences in the mitochondrial genome; these include 49 mononucleotides, 22 dinucleotides, 9 trinucleotides, 42 tetranucleotides, 1 pentanucleotide, and 1 hexanucleotide (**Figure 2**; **Supplementary Table S4, S5**). Monomeric and tetrameric repeats were found to be the most prevalent types of SSRs, accounting for 39.52% (49) and 33.87% (42) of all SSRs, respectively (**Supplementary Table S5**). Among the monomeric SSRs, A/T repeats constituted 97.96% (48) of the total count (**Supplementary Table S5**). Additionally, AAAG/CTTT repeats accounted for 23.81% (10) of the tetrameric SSRs, and AG/CT repeats represented 63.64% (14) of the dimeric SSRs (**Supplementary Table S5**). The majority of these repetitive sequences (111) were found to be located within the intergenic spacers (IGS), whereas introns contained 11 SSRs, and two were found in the open reading framework of *nad1* and *rps3*, respectively (**Supplementary Table S4**). These diverse SSR motifs offer numerous potential molecular markers for identifying and genetically analyzing *L. corniculatus*.

**Figure 3:**
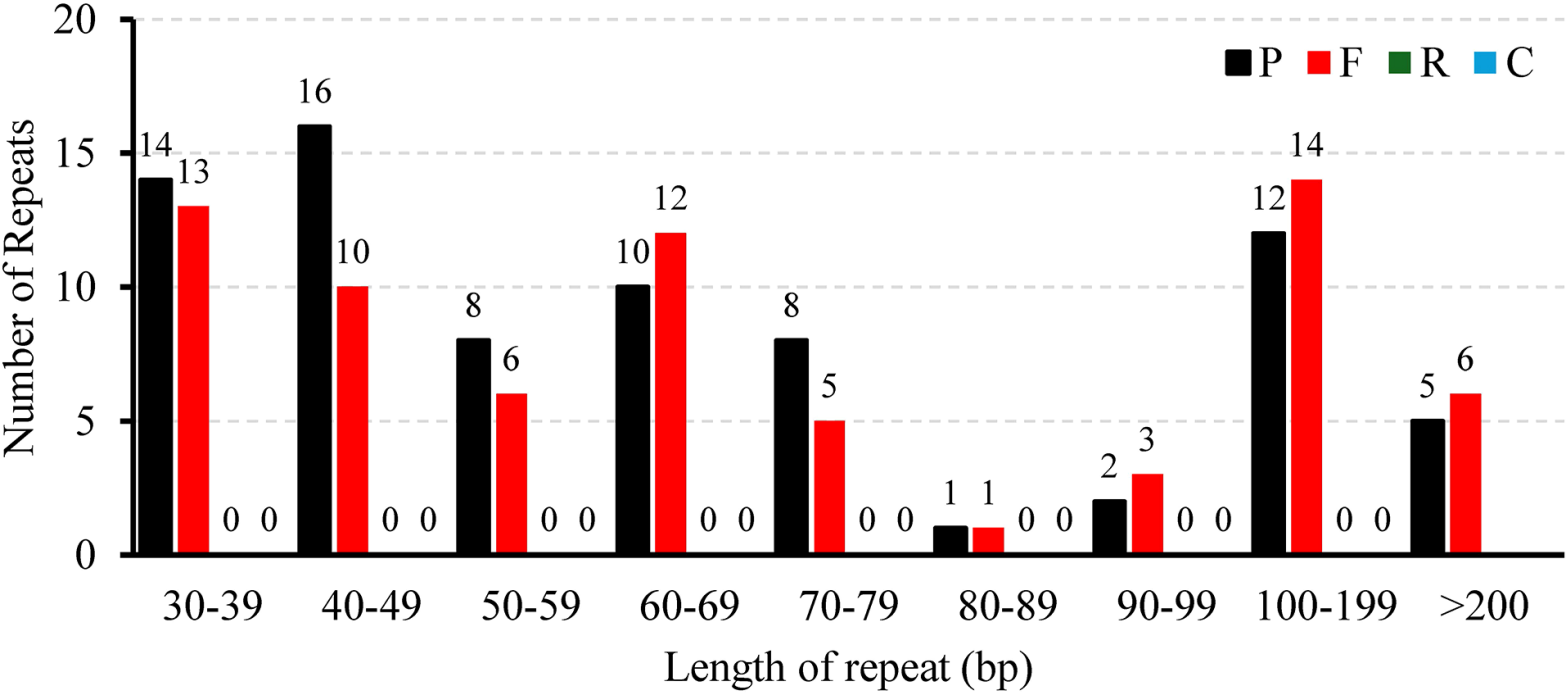
The length distribution of different types of repeats dispersed throughout the *Lotus corniculatus* mitochondrial genome.

**Figure 4:**
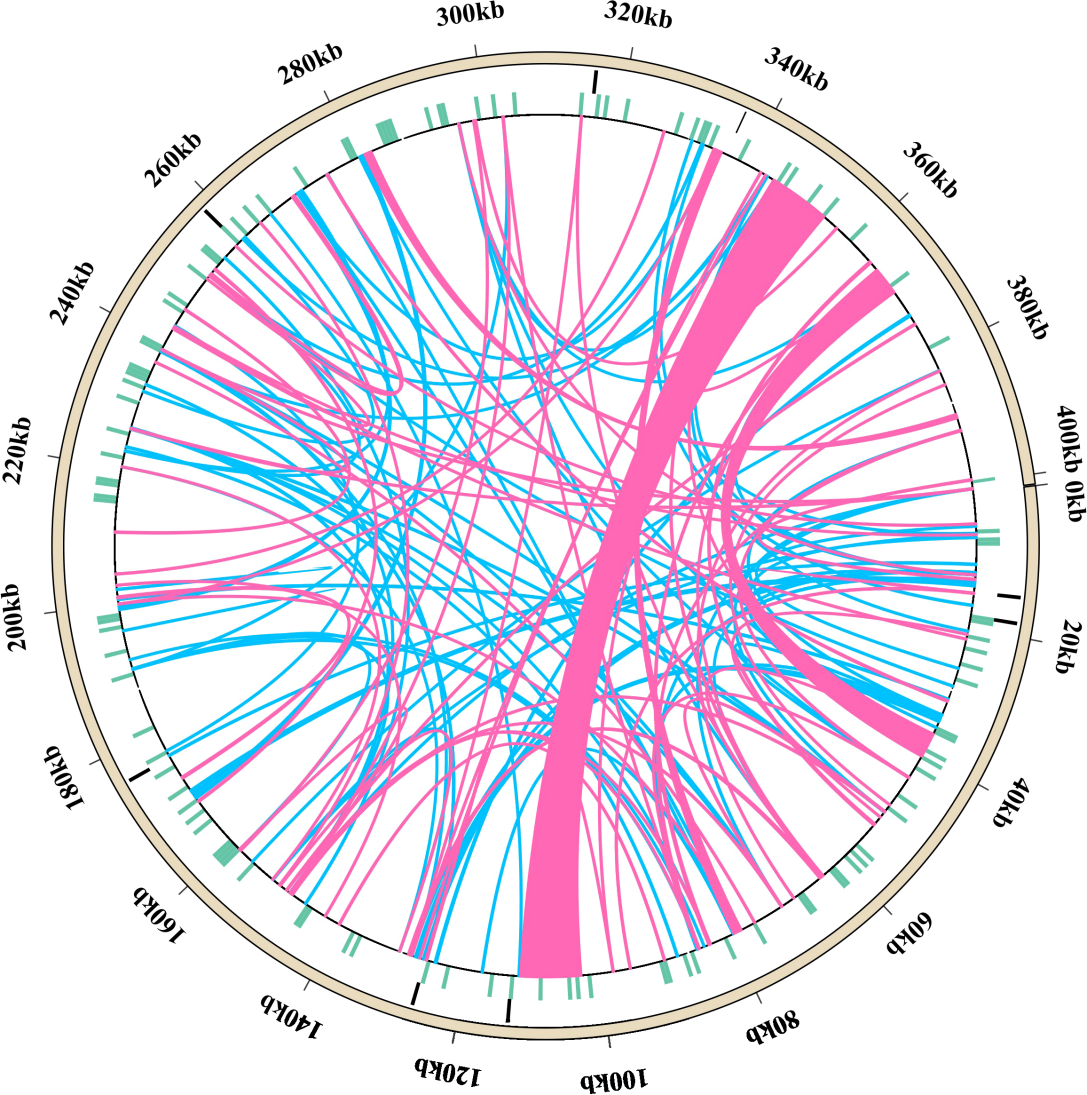
Diagram showing the distribution of repeat sequences in the *Lotus corniculatus* mitochondrial DNA. Tandem repeats, simple sequence repeats (SSRs), scattered repeats, and mitochondrial genome sequences are represented by circles, going from the outside to the inside. The pink arc indicates 70 forward repeats and the blue arc indicates 76 palindromic duplicates.

A total of 146 interspersed repeats were identified in the *L. corniculatus* mitogenome; these include 70 forward duplicates (F, 47.95%) and 76 palindromic duplicates (P, 52.05%). No reverse (R) or complement (C) duplications were detected (**Figure 3**). The repeat lengths are dispersed unevenly. The lengths of majority (92.47%) of the repeats are between 30 and 200 bp, with 11 (7.53%) repeats having length greater than 200 bp and 3 repeats being larger than 1 kb in length. Among these, the largest repeat has a length of approximately 9 kb (8,946 bp). Most of the scattered repeats were IGS-located sequences and IGS-located repeats (96, 65.75%), followed by IGS-located sequences and extron-located repeats (23, 15.75%) (**Supplementary Table S6**). In **Figure 4**, the pink arcs in the 340–360 kb and 100–120 kb regions are closely clustered, indicating a relatively dense distribution of corresponding positive repeats. Conversely, the dispersed arrangement of most of the blue arcs suggests a relatively scattered distribution of palindromic repeats (**Figure 4**).

The *L. corniculatus* mitochondrial genome contains eight tandem repeats, with 6 repeat sequence being 100% identical, and the remaining two showing more than 80% similarity. The outermost circle in **Figure 4** depicts the approximate positions of the eight tandem repeats within the mitochondrial genome. These repeats vary in length from 3 to 36 bp and are all located in IGS. Notably, not all repeats repeat an integer number of times should be acknowledged, such as the TCA repeats exhibit a copy number of 8.3 (**Supplementary Table S7**).

### 3.3 Codon usage in PCGs

**Figure 5:**
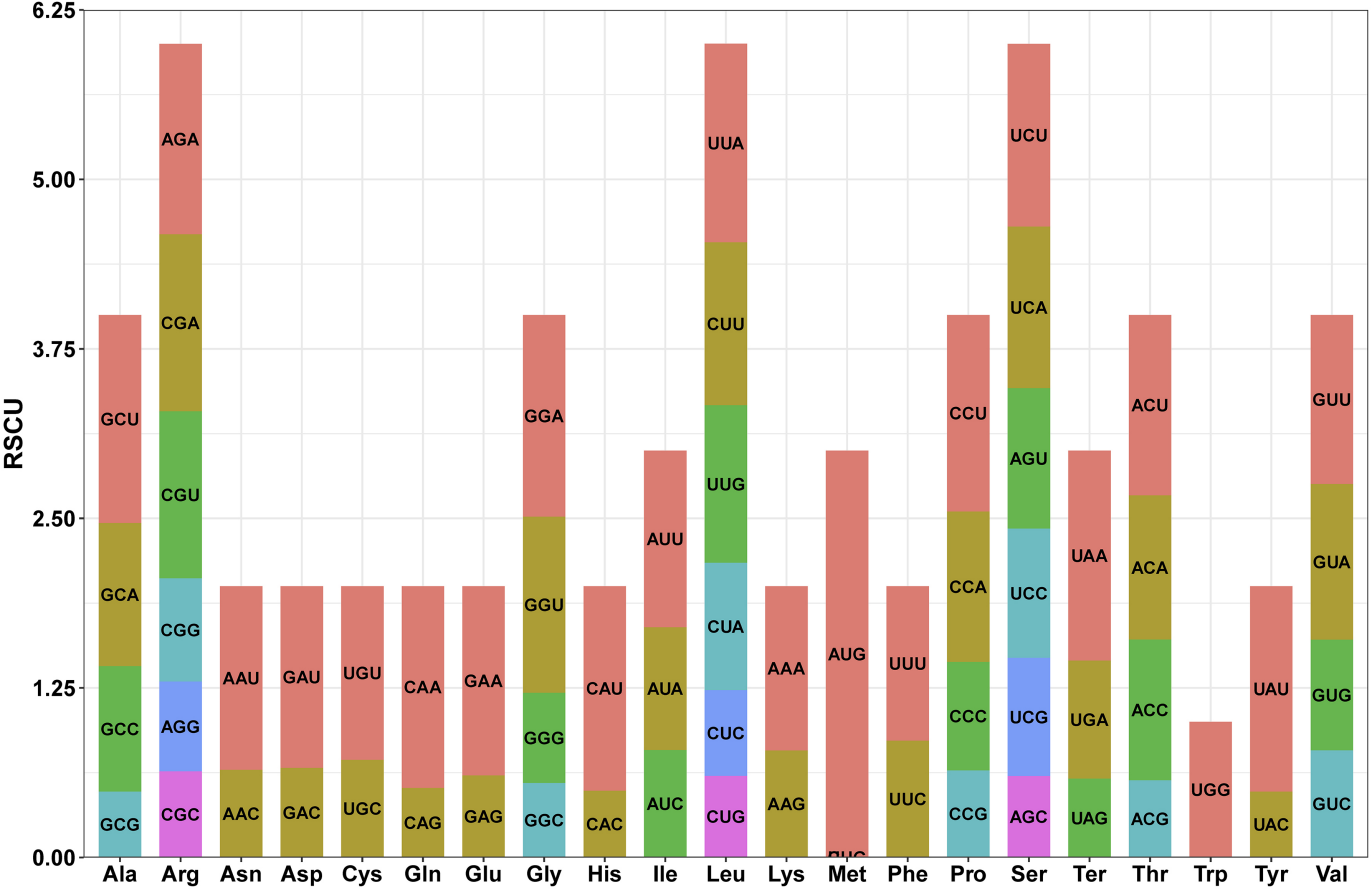
Relative synonymous codon use (RSCU) in the protein coding genes of the *Lotus corniculatus* mitochondrial genome. One color at a time, the codon for each amino acid correlates to that color.

Most PCGs in the *L. corniculatus* mitochondrial genome start with the ATG codon. However, the start codons of *nad1* and *nad4L* are ACG. Five types of stop codons terminate the PCGs—TAA in 16 genes (*atp1*, *atp4*, *atp8*, *atp9* (two copies), *cox1*, *nad1*, *nad2*, *nad3*, *nad4L*, *nad5*, *nad6*, *nad9*, *rpl16*, *rpl5*, and *rps4*); TGA in 8 genes (*ccmB*, *ccmFn*, *cob*, *cox2*, *cox3*, *nad4*, *rps10*, and *rps12*), TAG in 6 genes (*ccmC*, *matR*, *mttB*, *nad7*, *rps14*, and *rps3*), CAA in *atp6*, and CGA in *ccmFc* (**Figure 5**; **Supplementary Table S2**). There PCGs in the *L. corniculatus* mitochondrial genome contain a total of 9,579 codons. Of the total codons, 31 are stop codons. The most frequently used codon is UUU encoding Phe, accounting for 3.76% (360) of all codons, whereas the least used codon is UGC encoding Cys, accounting for only 0.52% (50) of all codons. The highest number of codons (1008) encoding Leu, followed by those encoding Ser (881) and Ile (746). The number of Cys-encoding codons is the lowest at 139 (**Table 2**).

**Table 2:** Codon count statistics for the protein-coding genes of the *Lotus corniculatus* mitochondrial genome PCGs.

Codon	Count	Codon	Count	Codon	Count	Codon	Count
UAA(*)	16	GGC(G)	90	AUG(M)	262	AGU(S)	152
UAG(*)	6	GGG(G)	109	AAC(N)	99	UCA(S)	175
UGA(*)	9	GGU(G)	213	AAU(N)	208	UCC(S)	140
GCA(A)	161	CAC(H)	58	CCA(P)	147	UCG(S)	128
GCC(A)	141	CAU(H)	178	CCC(P)	106	UCU(S)	198
GCG(A)	74	AUA(I)	225	CCG(P)	85	ACA(T)	131
GCU(A)	234	AUC(I)	197	CCU(P)	192	ACC(T)	128
UGC(C)	50	AUU(I)	324	CAA(Q)	198	ACG(T)	70
UGU(C)	89	AAA(K)	235	CAG(Q)	68	ACU(T)	164
GAC(D)	101	AAG(K)	153	AGA(R)	153	GUA(V)	173
GAU(D)	205	CUA(L)	158	AGG(R)	72	GUC(V)	119
GAA(E)	268	CUC(L)	106	CGA(R)	142	GUG(V)	123
GAG(E)	116	CUG(L)	101	CGC(R)	69	GUU(V)	188
UUC(F)	272	CUU(L)	202	CGG(R)	83	UGG(W)	143
UUU(F)	360	UUA(L)	246	CGU(R)	134	UAC(Y)	74
GGA(G)	244	UUG(L)	195	AGC(S)	88	UAU(Y)	231

The asterisk (*) denotes a stop codon.

### 3.4 Prediction of RNA editing sites

**Figure 6:**
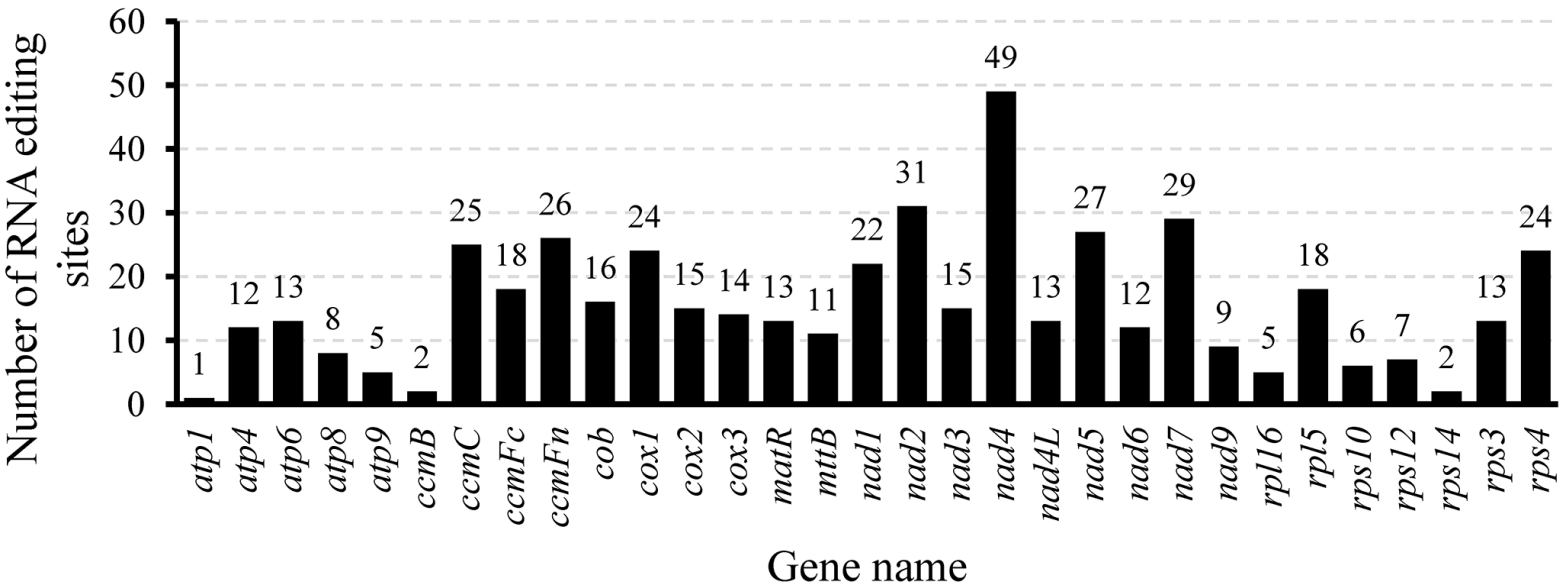
Distribution of RNA editing sites in protein-coding genes of the *Lotus corniculatus* mitochondrial genome.

**Figure 7:**
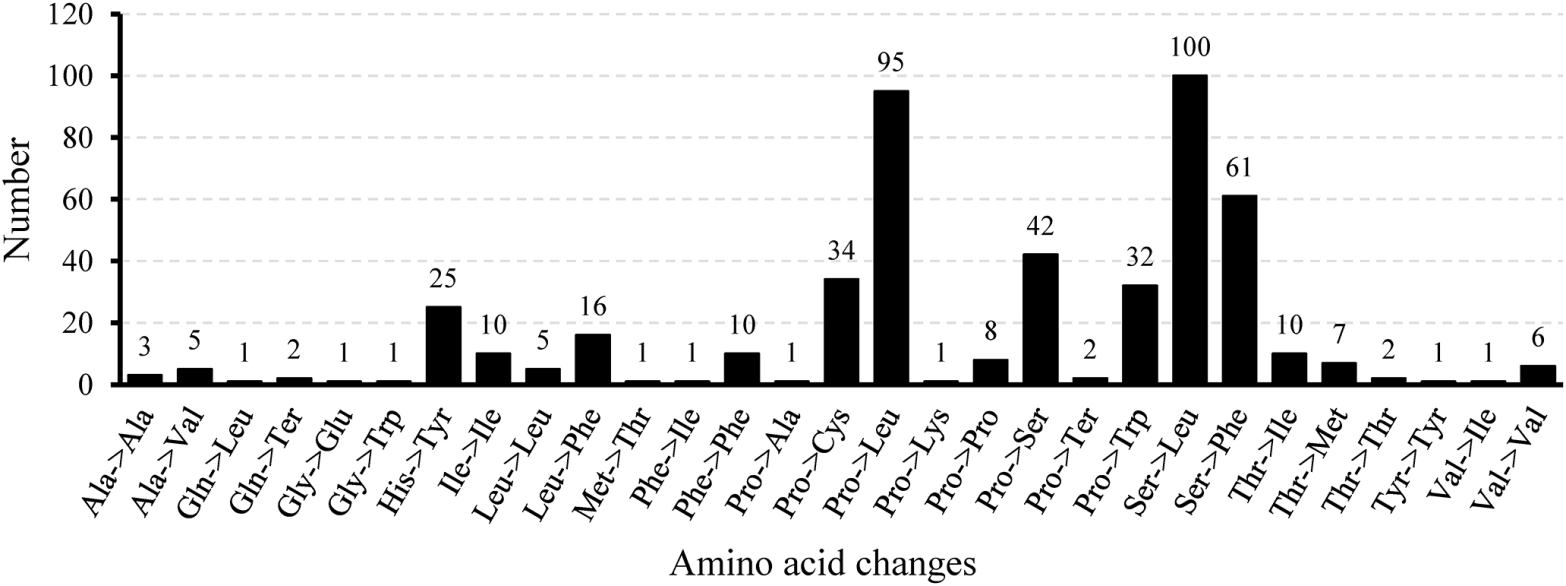
Frequency of amino acid changes induced by RNA editing in the *Lotus corniculatus* mitochondrial genome.

Mitochondrial gene expression is effectively regulated through RNA editing mechanisms (Gray, 2009). Zhang et al. (2024) counted the number of RNA editing sites in ten legume species and found that they ranged from 448 to 504. In this study, 32 CDS in the mitochondrial genome of *L. corniculatus* contained a total of 485 RNA editing sites, which was consistent with the statistical data of the aforementioned species, and was in the range of 448 to 504. Among the RNA editing sites of *L. corniculatus* mitochondrial genome, 42 (8.66%) were synonymous substitution sites and 443 (91.34%) were non-synonymous substitution sites. In addition, 467 C-to-T edits of RNA sites were found. The RNA-editing sites in *atp6* and *ccmFc* aid the development of stop codons. Similarly, initiation codons in *nad1*, *nad4L*, and *rps1* were introduced at three RNA editing sites. NADH dehydrogenase genes had a significant number of RNA editing sites (207), with *nad4* (49) having the highest number and *nad2* (31) having the second highest number. In *rps1*, a single edit of the RNA site was expected. Two editing sites were predicted in *rps14* and *ccmB*. Only 41 (8.45%) RNA editing sites were predicted to be located at the third codon position, whereas the maximum number of the RNA editing sites (161, 33.20%) were found at the first and second positions (283, 58.35%) (**Figure 6**; **Supplementary Table S8**). We identified 29 different amino acid conversions at these RNA-editing sites. Eight synonymous substitutions were identified, namely, Ala to Ala (3), Ile to Ile (10), Leu to Leu (5), Phe to Phe (10), Pro to Pro (8), Tyr to Tyr (1), Thr to Thr (2), and Val to Val (6). The number of nonsynonymous substitutions was 21. The replacement of Ser with Leu accounted for the highest number (100) of nonsynonymous substitutions, and was followed by replacement of Pro with Leu (95). Eight nonsynonymous replacements were identified, all of which occurred only once. In 100 (20.66%) and 95 (19.63%) instances, respectively, Ser and Prol were converted to Leu, with the highest frequency (**Figure 7**).

### 3.5 Identification of MTPTs

**Figure 8:**
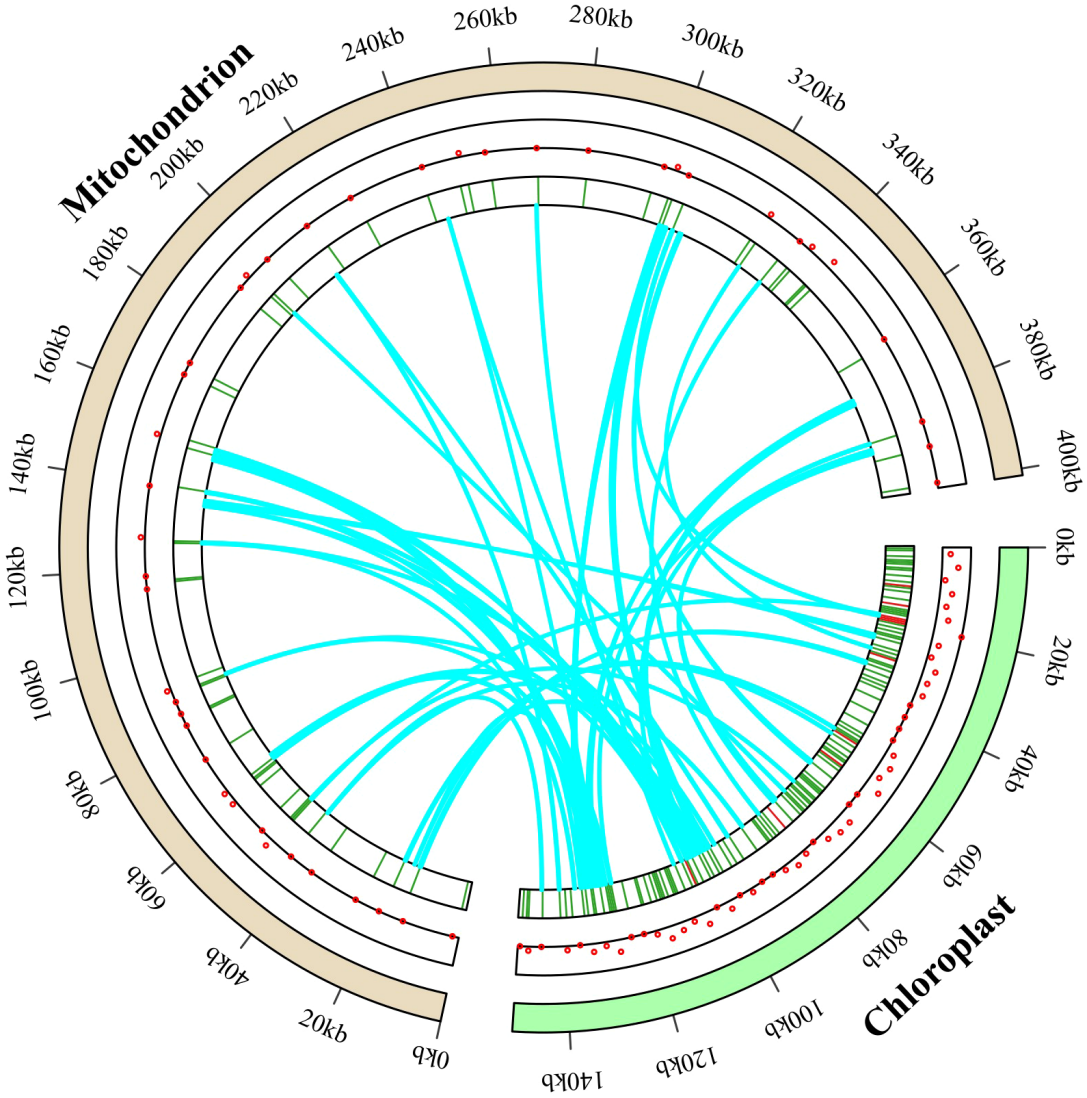
Gene transfer between the entire *Lotus corniculatus* chloroplast and mitochondrial genomes. The distribution of migratory genes is shown by the red dots in the central circle. The exact position of the migratory genes is indicated in the inner circle. The paths of gene transfer from the photosynthetic genome to the mitochondrial genome are depicted by the blue arc.

Fifty-seven identical fragments, totaling 16,211 bp in length and making 4.04% of the *Lotus corniculatus* mitochondrial genome, were found among the mitochondrial and chloroplast genomes based on the analysis of sequence similarity. The size of these fragments spanned from 42 to 1,245 bp. The chloroplast genome contains three fragments greater than 1,000 bp in length, with the longest *rps12* and *rrn23* sequences (1,245 bp) transferred to IGS of *atp8* and *trnK-UUU* in the mitochondrial genome. The migrating sequences mainly move from the intergenic regions of the chloroplast genome, tRNA genes, and PCGs to the IGS and tRNA genes in the mitochondrial genome. The *L. corniculatus* chloroplast genome contains eight *rrn16* sequences inserted into the mitochondrial genome, primarily integrated into the IGS region and *rrn18*. Additionally, the chloroplast genome harbors 14 *rrn23* sequences, which are predominantly incorporated into the IGS and *rrn26* regions. The chloroplast genome transfers to homologous segments of the mitochondrial genome, mostly nonfunctional, to IGS regions. However, a subset of genes including six *rrn26*, four *rrn18*, one *rps12* and one *nad5* are transferred from chloroplasts to mitochondria while maintaining their functional integrity. Additionally, five tRNAs are transferred from chloroplasts and retain their tRNA functionality (**Figure 8**; **Supplementary Table S9**).

### 3.6 Phylogenetic analysis of *Lotus corniculatus*

**Figure 9:**
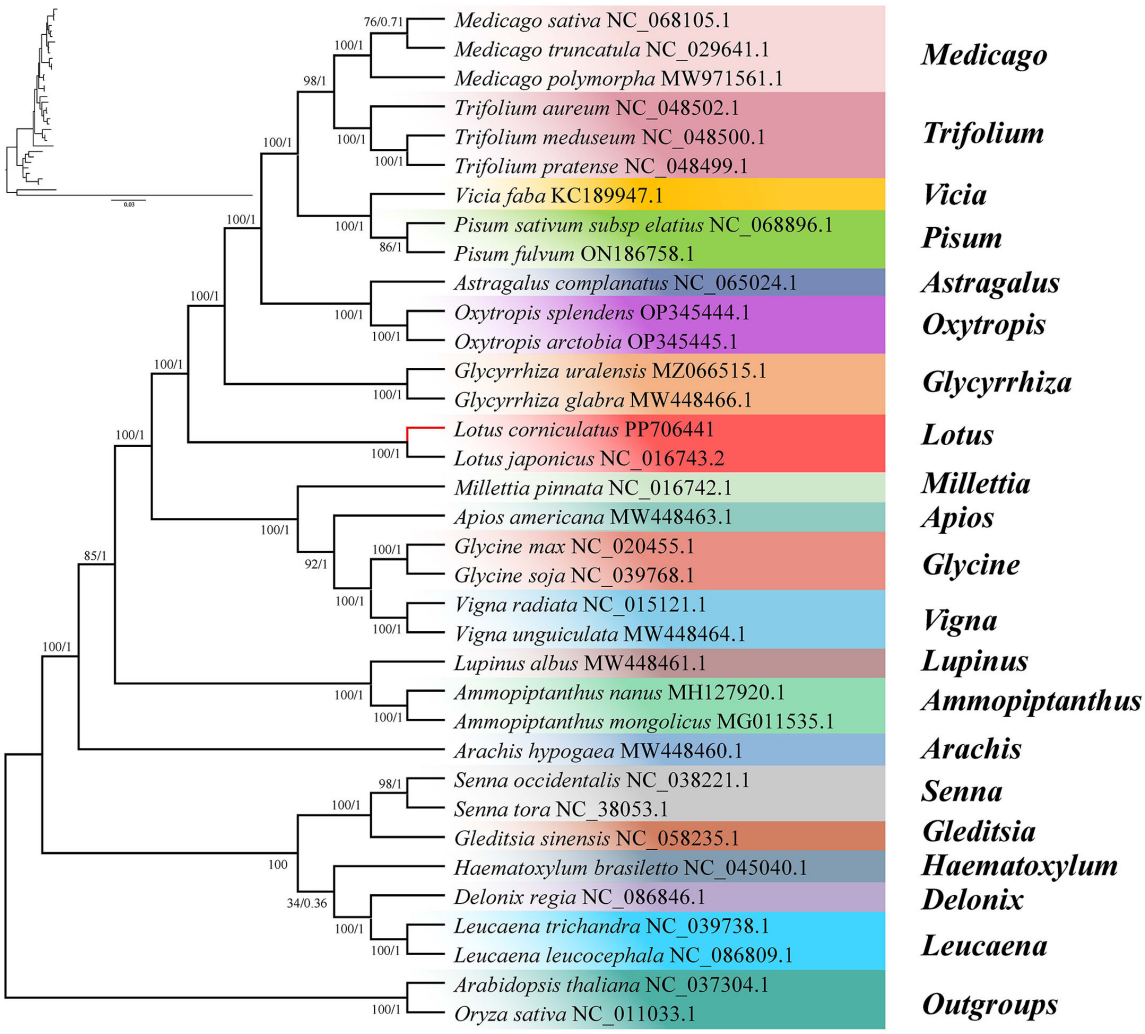
Phylogenetic relationship between *Lotus corniculatus* and 32 legume species. *Oryza sativa* and *Arabidopsis thaliana* are the outgroups. The value of each node represents the maximum likelihood bootstrap-support value and the Bayesian posterior probability. Color indicates genus.

We constructed phylogenetic trees using common genes of 35 species from four subfamilies, namely Papilionoideae, Caesalpinioideae, Oryzoideae, and Camelineae. *Oryza sativa* and *Arabidopsis thaliana* were selected as the outgroup taxa (**Figure 9**). According to the ML phylogenetic tree analysis, the majority of nodes exhibited a bootstrap support value >60%, with only one node having a value of 34%. Notably, 25 nodes displayed a bootstrap support degree of 100%. The BI analysis revealed that the Bayesian posterior probabilities for nearly all nodes were 1, with only two nodes having probabilities of 0.71 and 0.36, respectively, indicating the high reliability of the constructed phylogenetic tree. The ML and BI phylogenetic trees provided compelling evidence supporting a close evolutionary relationship between *L. corniculatus* and *L. japonicus*. According to the phylogenetic tree, the mitochondrial genomes of all taxa were classified into two branches based on their evolutionary relationships. The first branch comprised 15 genera of the Papilionoideae subfamily, namely *Ammopiptanthus*, *Apios*, *Arachis*, *Astragalus*, *Glycine*, *Glycyrrhiza*, *Lotus*, *Lupinus*, *Medicago*, *Millettia*, *Oxytropis*, *Pisum*, *Trifolium*, *Vicia*, and *Vigna*. These included *Arachis hypogaea*, which formed a distinct monophyletic clade, whereas the remaining species formed another sublineage. The second clade comprised five species of Caesalpinioideae and was further divided into two subclades—*Gleditsia* and *Senna* constituted one secondary clade, whereas *Delonix*, *Haematoxylum*, and *Leucaena* formed the other.

### 3.7 Collinearity analysis

**Figure 10:**
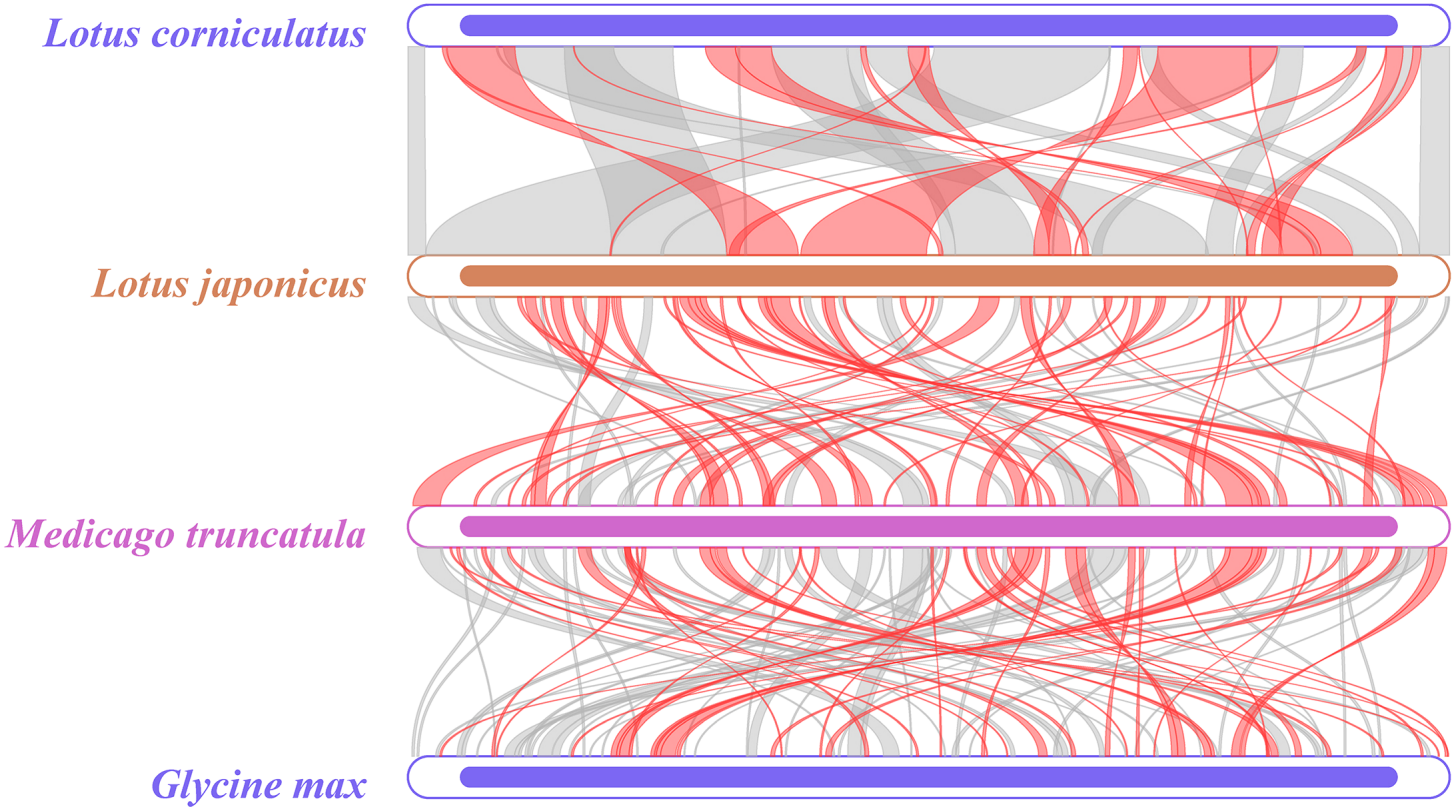
Multicollinearity map of the mitochondrial genomes of four leguminous species. Homologous sequences are represented by arcs, with gray arcs indicating positive homologous regions and red arcs denoting reverse homologous regions.

Collinear analysis can be used to study the evolutionary relationships of species by examining the relationships between homologous genes or sequence alignments. A comparison was conducted, comparing *L. japonicus* and *M. truncatula*, and *M. truncatula* and *Glycine max*. The range of highly homologous regions of the mitochondrial genome between *L. corniculatus* and *L. japonicus* was found to be wider (**Figure 10**). Notably, the length of the homologous region of *L. corniculatus* is 347,909 bp, accounting for 86.70% of the total length of its mitochondrial genome. The length of the homologous region of *L. japonicus* was 364,916 bp, which accounted for 95.81% of the total length of its mitochondrial genome (**Supplementary Table S9**). This suggests that *L. corniculatus* is the most closely related to *L. japonicus*.

## 4 Discussion

### 4.1 Characterization of the *L. corniculatus* mitogenome

In recent years, the advent of highly efficient, budget-friendly, and accurate genome sequencing and assembly tools such as Mitofiner (Allio et al., 2020) and PMAT (Bi et al., 2024) has enabled the successful assembly of an increasing number of complex plant mitochondrial genomes (Han et al., 2024; Liu et al., 2023, 2024). In the present study, we successfully integrated second- and third-generation sequencing techniques to assemble the mitochondrial genome of *L. corniculatus* and performed an extensive analysis of its characteristics, with the aim of enhancing our genetic understanding of this economically and medicinally important species. As of September 2024, the NCBI database documents the complete mitochondrial genomes of 96 legumes. Most of the reported legume mitochondrial genomes of leguminous plants range in size from 240 to 700 kb, with the mitogenome of *Leucaena trichandra*, with a length of 722,009 bp, being the exception (Kovar et al., 2018). The mitochondrial genome size of leguminous plants varies significantly. The Fabaceae family comprises six subfamilies (Cercidoideae, Detarioideae, Duparquetioideae, Dialioideae, Caesalpinioideae, and Papilionoideae). Among these, Papilionidae is the subfamily with the largest number of reported mitochondrial genomes. The reported mitochondrial genome size of Papilionoideae ranges from 370,000 to 550,000 bp. For instance, *Astragalus membranaceus* has a mitochondrial genome length of 398,048 bp (Zhang et al., 2024), *Caragana* sp*inosa* has a mitochondrial genome length of 378,373 bp (Zhou et al., 2023), and *Sophora koreensis Nakai* has a mitochondrial genome length of 519,841 bp (Ha et al., 2024). *L. corniculatus* belongs to the Papilionoideae subfamily. The mitochondrial genome of *L. corniculatus* assembled in this study has a size of 401,301 bp, indicating a significant expansion compared to the four *Trifolium* mitogenomes, which range from 294,911 to 348,724 bp (Choi et al., 2020). Analysis of repeat sequences revealed 278 repeats within the genome, comprising 17.53% of its total length, with long repeats (>1 kb) predominantly located in gene spacer regions. In contrast, repeats in the four *Trifolium* mitogenomes account for 6.6–8.6% of their respective genome lengths. Therefore, homologous recombination of repeated sequences may contribute to the observed genome expansion. However, genome size is influenced by factors beyond repeat content (Choi et al., 2020; Guo et al., 2017; Zhang et al., 2024). For instance, despite the *Vitis vinifera* mitochondrial genome being nearly 773 kb in size, its repeat content constitutes only 7% of the genome (Goremykin et al., 2009). Mitochondrial genome size variations are also shaped by the interplay of gene transfer and gene loss. Future research should involve larger sample sizes to elucidate how these factors directly impact plant evolution and adaptation.

One notable gene deletion in legumes is *cox2* (Nugent and Palmer, 1991). However, some statistical results suggest that *cox2* are present in some taxa, while absent in others (Adams et al., 1999). Nevertheless, as indicated in **Table 1**, the absence of *cox2* was not observed in *L. corniculatus* and it was also present in *L. japonicus* (Kazakoff et al., 2012). Therefore, *Lotus* can be tentatively identified as a genus in which no *cox2* deficiency exists. In angiosperms, the loss of ribosomal protein genes such as *rps1* is common during evolution; however, genes associated with respiration (*cox2* and *sdh3*) are rarely lost (Palmer et al., 2000). Nevertheless, the findings presented in this study demonstrate that both *rps1* and *sdh3* are lost in *L. corniculatus*, which deviates slightly from the aforementioned conclusions. In the process of evolution, gene transfer in plants may confer adaptive advantages in adaption (Neelapu et al., 2019; Ma et al., 2022). The transfer of organelle genome genes to the nuclear genome is very common (Chen et al., 2015a; Kleine et al., 2009; Ma et al., 2015; Martin, 2003; Pinard et al., 2019; Shahmuradov et al., 2003; Zhang et al., 2020b). For example, Zhang et al. (2020b) counted and analyzed transfer events from organelle genome sequences to nuclear genomes in more than 200 plants (Zhang et al., 2020b). When the ribosomal genes in the mitochondrial genome are transferred to nuclear genes or replaced by nuclear genes, the ribosomal genes cannot be expressed, resulting in the formation of pseudogenes. Therefore, the pseudogenization of certain ribosomal genes may be attributed to their transfer to the nuclear genome (Wang et al., 2024a). Another respiratory gene of *L. corniculatus*, *sdh4*, is a pseudogene (**Table 1**). In particular, the presence of *L. corniculatus sdh4* as a relatively intact pseudogene suggests that it may have undergone transfer to the host nuclear genome. The complete sequence of *sdh4* pseudogenes reveals the absence of large-scale deletions. However, the absence of necessary regulatory elements, signaling pathways, or synergies with other genes likely prevents proper expression or function, leading to pseudogenization (Wang et al., 2024b). Consequently, gene transfer appears to be the primary mechanism underlying the formation of relatively complete yet non-functional pseudogenes. Additionally, the high mutation rate inherent in mitochondrial genomes and functional redundancy may also contribute to the emergence of the *sdh4* pseudogene in *L. corniculatus*. The formation of pseudogenes not only exemplifies genome evolution but also provides valuable insights into genome dynamics and adaptability.

### 4.2 Codon preference and GC content of the *L. corniculatus* mitogenome

A deeper understanding of the plant organelle genome can be achieved through analysis of codon usage and GC content. The codon preference and GC content (45.15%) analyzed in this study for *L. corniculatus* were consistent with the highly similar patterns observed in the legume mitochondrial genome (Satrio et al., 2023; Yakovchuk et al., 2006). The utilization of codons significantly affects the evolutionary trajectory of the mitochondrial genome and can serve as a valuable tool for elucidating gene function, gene expression, and mRNA and protein abundance. In *L. corniculatus*, leucine was the predominant amino acid with the highest frequency of codon usage. Similarly, *Gleditsia sinensis*, a leguminous plant species, exhibits an increased preference for leucine in terms of its codon usage patterns. Furthermore, codon utilization influences mutations within genes, thereby facilitating phylogenetic association studies. The GC content of *L. corniculatus* was 45.15%, whereas that of *L. japonicus*, *Pongamia pinnata*, *Dalbergia odorifera*, *Apios americana*, *Glycine max*, *Glycine soja*, *Indigofera tinctoria*, *Millettia pinnata*, *Mucuna Pruriens*, *Phaseolus vulgaris*, *Vigna angularis*, *Vigna radiata*, and *Pisum sativum* was 45.4%, 45.0%, 45.1%, 44.98%, 45.03%, 45.03%, 44.73%, 45.00%, 45.28%, 45.11%, 45.19%, 45.11%, and 45.08% respectively (Hong et al., 2021; Kazakoff et al., 2012; Satrio et al., 2023; Yakovchuk et al., 2006). High GC content increases heat stability and extends cell life by regulating the moderate mutation rate of mitochondrial genome compared with that of the chloroplast genome. Thermal stability is crucial for the survival and adaptability of plants in environments with varying temperatures. In the context of global warming, increasing temperatures present novel challenges to plant viability (Seth and Sebastian, 2024). A plant mitochondrial genome exhibiting high thermal stability can more effectively adapt to elevated temperatures, thereby maintaining mitochondrial function and ensuring adequate energy supply and plant growth (Raza et al., 2024). By investigating the relationship between GC content and thermal stability within the plant mitochondrial genome, we can gain deeper insights into the evolutionary and adaptive mechanisms that enable plants to thrive in diverse environmental conditions. Therefore, future studies should systematically analyze the relationship between GC content and thermal stability using extensive mitochondrial genome data from legumes.

### 4.3 RNA Editing of the *L. corniculatus* mitogenome

Plant mitochondrial genomes experience extensive RNA editing, a process crucial for regulating gene expression. Notably, specific RNA editing sites vary across different plant species. For example, the mitochondrial genome of *Arabidopsis thaliana* (Unseld et al., 1997) contains 36 CDS with 441 RNA editing sites, whereas *Rehmannia chingii* mitochondrial genome (Han et al., 2024) harbors 47 CDS with 579 RNA editing sites. In the present study, we identified 485 RNA editing sites within 32 CDS regions of the *L. corniculatus* mitochondrial genome. The statistics for nine reported leguminous plants, including *Astragalus membranaceus*, *Astragalus complanatus*, *Caragana* sp*inosa*, *Glycyrrhiza glabra*, *Medicago sativa*, *Oxytropis arctobia*, *Pisum fulvum*, *Trifolium aureum*, and *Trigonella foenum-graecum*, revealed predicted RNA editing sites ranging from 448 to 504 (Zhang et al., 2024). It is noteworthy that in these leguminous plants, the gene containing the highest proportion of RNA editing sites is *nad4*. The protein encoded by the *nad4* gene is a critical component of mitochondrial respiratory chain Complex I. Respiration plays an indispensable role in plant energy metabolism, providing essential energy for growth, development, and various physiological activities (Ren et al., 2020). Consequently, proper expression and functionality of *nad4* are vital for plant survival. Given its significance, this gene has likely been subject to multiple regulatory mechanisms throughout evolution, with RNA editing being a prominent mode of regulation. RNA editing can modify the sequence of the *nad4* gene transcript, thereby influencing the structure and function of the encoded protein to meet diverse physiological demands (Lamattina and Grienenberger, 1991). However, direct studies on the proportion of *nad4* gene RNA editing sites across different growth stages in legumes remain limited. Future research should focus on conducting comprehensive transcriptome analyses at various growth stages in legumes to elucidate changes in the *nad4* gene RNA editing sites and further uncover its role in plant growth and development.

### 4.4 Gene transfer between organelle genomes

Intermediate gene transport refers to the intracellular transfer of genetic sequences between the genome, plasmid, and nucleus (Hao and Palmer, 2009). Integration of chloroplast genomic DNA fragments into mitochondrial genomes is a commonly observed phenomenon (Wei et al., 2022). We identified six coding sequences (*rbcL*, *accD*, *psaA*, *atpA*, *ycf2*, and *nadhF*) that migrate from *L. corniculatus* chloroplast genome to the mitochondrial genome. Notably, some of these genes have been annotated in the mitochondrial genome. We propose that other genes originating from chloroplasts may have undergone pseudogenization (Choi and Park, 2021). In flowering plants, transfer RNA (tRNA) genes frequently migrate from the chloroplast to the mitochondrial genome (Bi et al., 2016). Furthermore, we detected this phenomenon in *L. corniculatus* organelle genomes with *trnN*-*GUU* and *trnK*-*UUU* (**Supplementary Table S9**). Previous studies have shown that the *rps1* gene is completely absent from the mitochondrial genome of *L. japonicus*. Notably, its distant relatives *T. aureum*, *T. grandiflorum*, *T. meduseum* and *T. pratense* also exhibit complete deletion of the *rps1* gene in their respective mitochondrial genomes (Choi et al., 2020). This suggests that the functional transfer of *rps1* likely occurred at least twice within this family: once in *Lotus* and once in the ancestors of *Trigonella*, *Melilotus*, *Medicago* and *Trifolium* species (Choi et al., 2020). The deletion of the *rps1* gene in the mitochondrial genome of *L. corniculatus* further supports this claim. Consequently, during plant evolution, significant heterogeneity has arisen in mitochondrial genomes owing to active horizontal gene transfer events (Chen et al., 2020).

### 4.5 Mitochondrial phylogenomics in Fabaceae

In 2017, the Legume Phylogeny Working Group constructed a legume phylogenetic tree based on chloroplast *matK* sequence data that is the most well-sampled to date (about 91% of genera and 20% of species), and combined with morphological evidence, proposed a new taxonomic system for six subfamilies (Li et al., 2023). However, there are still many disputes and problems that cannot be solved satisfactorily. In this study, we constructed phylogenetic trees of 15 genera of Papilionoideae and 5 genera of Caesalpinioideae based on mitochondrial genomes. The systematic position of *L. corniculatus* in the leguminous family was determined. In conjunction with collinearity analysis, it can be concluded that *L. corniculatus* and *L. japonicus* exhibit a close phylogenetic relationship (**Figure 10**). The topological structure of this phylogenetic tree is consistent with that of reported legume phylogenetic trees, and it has very high branch support (Ha et al., 2024; Tian et al., 2021; Zhang et al., 2024; Zhou et al., 2023). It is notable that among the 15 genera of Papilionoideae in this research, the differentiation of *Arachis hypogaea* occurred earlier than that of the other species, and it has a relatively distant phylogenetic relationship with the species of the other 14 genera (**Figure 9**). The phylogenetic position of *Arachis hypogaea* is consistent with the legume system constructed based on the mitochondrial genome (Zhang et al., 2024; Choi et al., 2021), but not consistent with the legume system constructed based on the chloroplast genome (Ha et al., 2024). The reasons for this need to be further studied. Therefore, to construct more reliable phylogenetic relationships within legume subfamilies, more comprehensive analysis of genomic data and geographical aspects of legumes is needed.

## 5 Conclusions

We successfully completed the assembly and annotation of the mitochondrial genome of L. corniculatus, yielding a high-quality genomic sequence. The length of *L. corniculatus* mitochondrial genome was determined to be 401,301 bp, with a GC content of 45.15%. A total of 53 genes were encoded, including 32 CDS, 18 tRNAs, and 3 rRNAs. Additionally, we identified 146 scattered repeats, 8 tandem repeats, and 124 SSRs. Furthermore, RNA editing site prediction, codon preference, gene transfer, and phylogenetic analyses were conducted on *L. corniculatus* mitochondrial genome. These results have important ramifications for the development of molecular markers that should improve our understanding of the composition and evolution of the mitochondrial genome of *L. corniculatus* and would help scientists understand the evolution and taxonomy of Fabaceae.

## Data Availability

The datasets presented in this study can be found in online repositories. The names of the repository and accession number(s) can be found below: https://www.ncbi.nlm.nih.gov; Genbank accession number: PP706441.1; BioProject ID: PRJNA1216031; BioSample accession numbers: SAMN46423723; SRA accession numbers: SRR32134186, SRR32134187.
