# Supplementary material for: The complete mitochondrial genome and phylogenetic analysis of Lotus corniculatus (Fabaceae, Papilionoideae)
Source: Front Plant Sci. 2025 Mar 11;16:1555595. doi: 10.3389/fpls.2025.1555595 (PMC11933009; doi:10.3389/fpls.2025.1555595)
Supplement: Supplementary file 2 [file DataSheet1.docx]

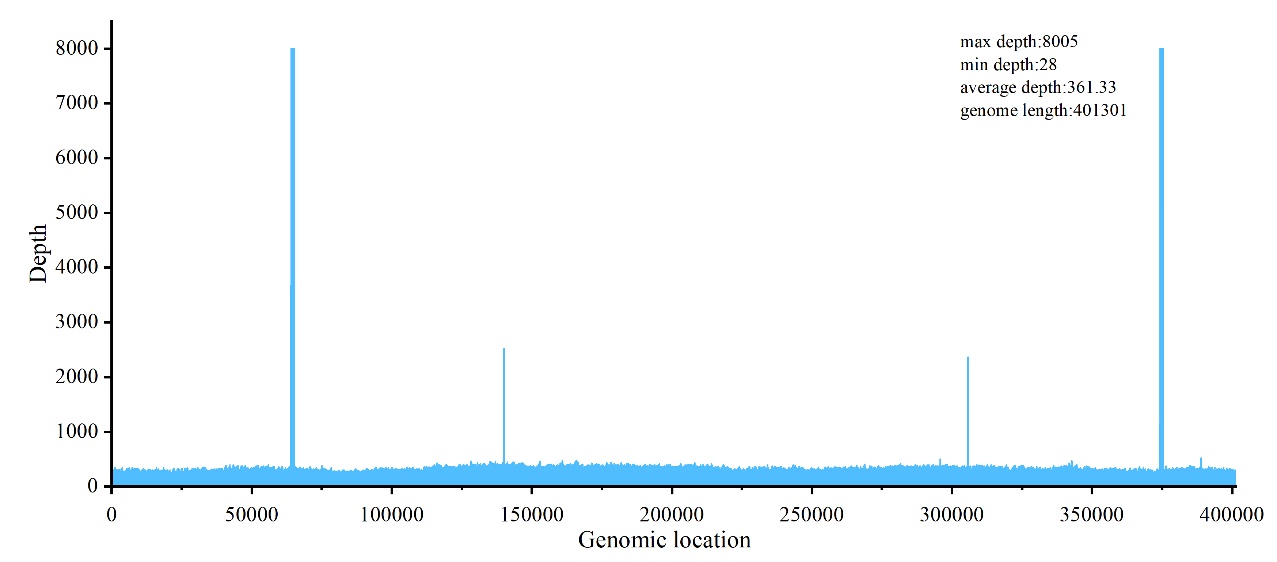


**Supplementary Figure S1** Coverage depth analysis of the Illumina NovaSeq 6000 data


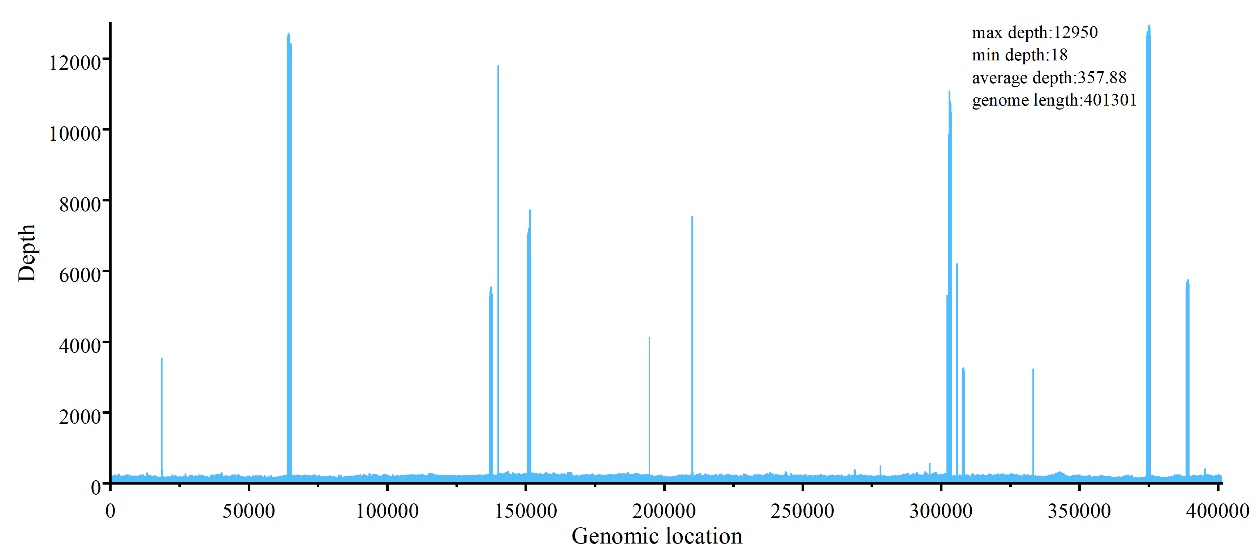


**Supplementary Figure S2** Coverage depth analysis of the Oxford Nanopore PromethION data
